# The Interaction between Dietary Fiber and Fat and Risk of Colorectal Cancer in the Women’s Health Initiative

**DOI:** 10.3390/nu8120779

**Published:** 2016-11-30

**Authors:** Sandi L. Navarro, Marian L. Neuhouser, Ting-Yuan David Cheng, Lesley F. Tinker, James M. Shikany, Linda Snetselaar, Jessica A. Martinez, Ikuko Kato, Shirley A. A. Beresford, Robert S. Chapkin, Johanna W. Lampe

**Affiliations:** 1 Division of Public Health Sciences, Fred Hutchinson Cancer Research Center, Seattle, WA 98109, USA; mneuhous@fredhutch.org (M.L.N.); ltinker@whi.org (L.F.T.); beresfrd@uw.edu (S.A.A.B.); jlampe@fredhutch.org (J.W.L.); 2 Department of Epidemiology, School of Public Health, University of Washington, Seattle, WA 98105, USA; 3 Division of Cancer Prevention and Population Sciences, Roswell Park Cancer Institute, Buffalo, NY 14263, USA; david.cheng@roswellpark.org; 4 Division of Preventive Medicine, University of Alabama at Birmingham, Birmingham, AL 35294, USA; jshikany@uabmc.edu; 5 Department of Epidemiology, University of Iowa, Iowa City, IA 52242, USA; linda-snetselaar@uiowa.edu; 6 Department of Nutritional Sciences, University of Arizona Cancer Center, Tucson, AZ 85724, USA; jam1@email.arizona.edu; 7 Department of Oncology and Pathology, Wayne State University, Detroit, MI 48201, USA; katoi@karmanos.org; 8 Program in Integrative Nutrition and Complex Diseases, Texas A&M University, College Station, TX 77843, USA; r-chapkin@tamu.edu

**Keywords:** butyrate, colorectal cancer, DHA, EPA, fat, fiber, omega-3, pectin

## Abstract

Combined intakes of specific dietary fiber and fat subtypes protect against colon cancer in animal models. We evaluated associations between self-reported individual and combinations of fiber (insoluble, soluble, and pectins, specifically) and fat (omega-6, omega-3, and docosahexaenoic acid (DHA) and eicosapentaenoic acid (EPA), specifically) and colorectal cancer (CRC) risk in the Women’s Health Initiative prospective cohort (*n* = 134,017). During a mean 11.7 years (1993–2010), 1952 incident CRC cases were identified. Cox regression models computed multivariate adjusted hazard ratios to estimate the association between dietary factors and CRC risk. Assessing fiber and fat individually, there was a modest trend for lower CRC risk with increasing intakes of total and insoluble fiber (*p-trend* 0.09 and 0.08). An interaction (*p* = 0.01) was observed between soluble fiber and DHA + EPA, with protective effects of DHA + EPA with lower intakes of soluble fiber and an attenuation at higher intakes, however this association was no longer significant after correction for multiple testing. These results suggest a modest protective effect of higher fiber intake on CRC risk, but not in combination with dietary fat subtypes. Given the robust results in preclinical models and mixed results in observational studies, controlled dietary interventions with standardized intakes are needed to better understand the interaction of specific fat and fiber subtypes on colon biology and ultimately CRC susceptibility in humans.

## 1. Introduction

Colorectal cancer (CRC) is the third most common type of cancer in the U.S., accounting for roughly 8% of new cancer cases and 8% of all cancer deaths in 2015 [1]. Lifestyle factors such as diet, physical activity, and body weight contribute substantially to CRC, and it is generally held that risk could be greatly reduced through dietary modification [2]. Dietary fiber and fat are two of the most well-studied dietary components in this regard [3]. Although many case-control studies have found inverse associations between fiber and fat intakes and CRC [4], data from epidemiologic studies are mixed [4,5,6,7,8,9,10,11,12,13,14,15,16,17,18,19,20,21,22,23,24,25], possibly due to differences in intake assessment, the type of fiber or fat analyzed, and residual confounding by other lifestyle and dietary factors. More recent studies have attempted to control for dietary confounders such as folate, alcohol, and red meat consumption, yet the contradictions in outcomes persist [7,15,26,27]. Finally, within the Women’s Health Initiative (WHI) Dietary Modification Trial, a low fat dietary pattern intervention which included a focus on increasing servings of whole grains, vegetables, and fruits, was found to have no significant benefit on CRC incidence over an average of 8.1 years [28]. 

There is a growing recognition that dietary exposures are complex with synergistic and antagonistic effects between different dietary components contributing to chronic disease risk. Interactions between dietary constituents and microbial metabolites may also partially explain differences in population-level study outcomes [29,30]. In contrast to the equivocal data from observational studies in humans—in which each dietary component is evaluated separately—strong evidence for a combined effect of the subtypes of fat and fiber in relation to reduced colon tumorigenesis has been demonstrated in preclinical animal models [31,32,33,34,35]. Gut bacteria ferment dietary fiber (e.g., pectins, a type of fermentable soluble fiber) to butyrate [36,37,38,39,40,41,42,43,44,45,46,47,48,49], a potent histone deacetylase (HDAC) inhibitor [29,30] associated with reduced CRC risk [50,51,52]. Omega 6 (*n*-6) polyunsaturated fatty acids (PUFA), commonly found in refined vegetables oils, processed and fast foods, and red meat, typical in Western diets [53], and omega 3 (*n*-3) PUFA in fish oils [54] are structural precursors of eicosanoids; those derived from n-6 are mainly pro-inflammatory, whereas those produced from *n*-3 tend to have opposing effects [55,56]. Given the strong correlation between inflammation and CRC [56], higher intakes of long-chain *n*-3 PUFA provide biologic validity for a chemoprotective effect [57].

Preclinical research suggests that the combined effects of *n*-3 PUFA in fish oils (e.g., docosahexaenoic acid (DHA) and eicosapentaenoic acid (EPA)) and butyrate from fermentable fiber (e.g., pectin) may work in concert to enhance the chemopreventive potential of either dietary component alone, primarily by increasing apoptosis [31,33,34,58,59,60,61,62]. While other factors, such as low dietary intakes of fiber and certain fatty acid subtypes, may contribute to the mixed results in humans, the lack of evaluation of the interaction between subtypes of dietary fat and fiber, i.e., pectins in soluble fiber and marine-derived *n*-3 PUFA, may also explain why the chemoprotective effects of fiber and fat have not been consistently detected in prospective cohort studies. Therefore, our objectives for this study were to determine the associations between the individual and combinations of subtypes of fiber (soluble, and pectins specifically, which leads to the production of butyrate, vs. insoluble) and fat (*n*-3, and DHA + EPA, specifically, vs. *n*-6), and risk of CRC in the WHI. 

## 2. Materials and Methods

### 2.1. Study Population

The WHI is a large prospective study focused on understanding preventive strategies for major chronic diseases in postmenopausal women [63]. WHI includes both a multi-component randomized Clinical Trial (CT) and an Observational Study (OS). The CT (*n* = 68,132) was actually three overlapping randomized controlled trials evaluating combined hormone therapy or estrogen alone versus placebo (HRT), a low-fat dietary pattern versus comparison (Dietary Modification), and calcium and vitamin D supplementation versus placebo (CaD), on the risk of breast and colorectal cancers, coronary heart disease, and osteoporotic fractures. The OS (*n* = 93,676) examines a broader range of lifestyle, health, and risk factors and risk of common chronic diseases in postmenopausal women initially interested in but ineligible for one or more of the clinical trials. Postmenopausal women aged 50–79 years were recruited from the general population at 40 different Clinical Centers between 1993 and 1998, with trial follow-up information and outcome data available through 2005, and post-trial follow-up information and outcome data for these analyses available through September, 2010, the end of the first WHI Extension Study. All participants in the CT and OS were included in the present analysis except women who were randomized to the intervention arm of the Dietary Modification trial (*n* = 19,541). Further details on recruitment, study design, and baseline measures have been published elsewhere [63,64,65,66]. Institutional review board approval was obtained from all clinical sites, and women provided written informed consent prior to participation and for follow-up in the Extension period. The WHI ClinicalTrials.gov identifier is NCT00000611.

### 2.2. Data Collection and Dietary Assessment

Detailed information on demographic characteristics, medical, reproductive and familial CRC history, and lifestyle factors were collected at baseline. Weight and height were assessed with a standardized protocol using a calibrated balance beam scale and stadiometer, respectively, and body mass index (BMI) was computed as weight in kg/height in m^2^. Physical activity was assessed by computing average weekly metabolic equivalents of moderate and vigorous leisure-time physical activity. 

Dietary intake over the past three months was measured from the WHI food frequency questionnaire (FFQ) [67] using a standardized protocol at baseline for all women, and at year one for Dietary Modification CT participants. Follow-up FFQs were completed in years two through nine for a rotating proportion (33%) of Dietary Modification CT participants. The baseline FFQ data were used for the current study. The FFQs contained 122 food and food group items, and 19 adjustment questions, the majority of which ask about food practices pertaining to types and amounts of added fats (e.g., fat added at the table and in cooking), and three summary questions on food purchasing and preparation methods [67]. The WHI nutrient database was derived from Nutrition Data Systems for Research (NDS-R, version 2005, Nutrition Coordinating Center, University of Minnesota, Minneapolis, MN, USA). The database has over 140 nutrient values including total fat, total PUFA, *n*-6 and *n*-3 PUFA, total DHA and EPA from food sources; and total, insoluble and soluble fiber, and pectins. 

### 2.3. Colorectal Cancer Case Ascertainment

Clinical outcomes, including cancer diagnoses, were updated annually in the OS and semi-annually in the CT until 2005 when the outcomes were thereafter reported annually using mail or telephone questionnaires. Self-reports of CRC were verified by trained physician adjudicators at the Clinical Centers who reviewed medical records and pathology reports [68]. All CRC diagnoses were then confirmed by blinded review at the WHI Clinical Coordinating Center at the Fred Hutchinson Cancer Research Center. 

### 2.4. Statistical Analysis

Exclusions were made for women who reported history of colon or rectal cancers prior to baseline enrollment or who were missing these data (*n* = 946). Further exclusions were made for women with FFQs containing incomplete information, or data that suggested biologically implausible daily energy intakes of <2512 or >20,930 kJ (*n* = 4649), or extreme BMIs (<15 or >50 kg/m^2^; *n* = 2069). Finally, women who were missing data for smoking, physical activity, and education variables combined (*n* = 9), or were lost to follow up (*n* = 577), were excluded, leaving a sample of 134,017 for analysis. 

Cox proportional hazards models were used to estimate hazard ratios (HR) and 95% confidence intervals (95% CI) for the association of fiber (total, insoluble, soluble, and pectins) and fat (total, *n*-6, *n*-3, and DHA + EPA) intakes and CRC risk. Dietary variables were evaluated as separate main effects, as quintiles of intake, using the lowest quintile as the reference group. Quintile assignments of dietary variables from the baseline FFQ were calculated based on the distribution in non-cases. Tests for trend were performed in a separate Cox model treating quintiles 1–5 as linear. Fat variables were analyzed both as g intake/day with energy adjusted in the models, and as % energy. As the results for both were similar (data not shown) we present only fat as g intake/day. Interactions (a total of seven determined a priori: total fat × total fiber; insoluble fiber × *n*-6 PUFA; soluble fiber × *n*-3 PUFA; insoluble fiber × *n*-3 PUFA; soluble fiber × DHA + EPA; pectins × *n*-3 PUFA; pectins × DHA + EPA) were assessed by adding the multiplicative interaction term between linear quintiles of dietary fat and fiber variables to the final multivariate model. For the significant interaction of soluble fiber and DHA + EPA, post-hoc HRs and 95% CIs were computed for each quintile comparison for each of the 25 fat × fiber groups. 

All models were adjusted for established or suggestive risk factors for CRC: (age (years, continuous); family history of CRC (yes/no); red and processed meat consumption (g/day, continuous); BMI (kg/m^2^, continuous); leisure physical activity (total metabolic equivalent-h/week, continuous); smoking (current or past/never); alcohol use (g/day, continuous); current use of non-steroidal anti-inflammatory drugs (NSAIDs; yes/no); folate (food plus supplements; µg dietary folate equivalents/day, continuous); calcium (food plus supplements; mg/day; continuous)) [69,70], and confounders that altered point estimates for dietary outcome measures by more than 10%: total energy intake (kcal/day, continuous); history of screening colonoscopy (yes/no); education level (high school; technical school or some college; college grad or post); ever use of menopausal hormones (yes/no); and study component (OS, CT) and CT randomization assignment (HRT, Dietary Modification or CaD). We created a “missing” category for covariates with data missing for <0.5% of the participant population (NSAIDs, *n* = 91, smoking, *n* = 1737, education level, *n* = 1005, and colonoscopy screening, *n* = 4736), and imputed the median values for physical activity (*n* = 5230) to reduce the number of participants who would be dropped from the analysis. All tests for individual dietary factors were two sided and statistical significance was set at *p* < 0.05. Tests for interaction were adjusted for multiple testing using Bonferroni correction (0.05/7 tests conducted = *p* < 0.007). All analyses were conducted using Stata (v14.1; StataCorp, College Station, TX, USA).

## 3. Results

The distribution of baseline participant characteristics and dietary intake for CRC cases and non-cases are given in Table 1. During a mean follow up of 11.7 years (±3.5; range: 0.02–17.5 years), a total of 1952 CRC cases were reported. Women with CRC appeared less likely to have ever used hormone therapy and consumed less calcium. Intakes of all other dietary variables were very similar in cases and non-cases.

Table 2 provides data on associations of total, insoluble, and soluble fiber, and pectins, with CRC risk. When assessing fiber as a main effect, higher versus lower total soluble and insoluble fibers were associated with modest, but non-significant lower risk of CRC. There was a suggestion of inverse linear trends for total and insoluble fiber, (*p-trend* = 0.09 for total fiber; *p-trend* = 0.08 for insoluble fiber). 

The associations of total fat, *n*-6 PUFA, *n*-3 PUFA, and EPA + DHA with CRC risk were null with most HRs near the null value of 1.0 (Table 3). 

We next examined the interaction of fat and fiber subtypes in relation to CRC risk. The interaction between DHA + EPA and soluble fiber was statistically significant (*p-interaction* = 0.01), with a significant decreased risk of CRC with increasing DHA + EPA intake among those in the lowest quintile of soluble fiber intake (*p-trend* = 0.02; Table 4) and a borderline increased risk of CRC with increasing DHA + EPA among those in the highest quintile of soluble fiber (*p-trend* = 0.07; Table 4). There was a marginal statistically significant association between pectins and DHA + EPA (*p* = 0.05), with a similar pattern of attenuation, whereby the protective effects of higher intakes of pectins were no longer apparent with higher intakes of DHA + EPA (data not shown). However, these interactions were no longer significant when adjusted for multiple comparisons. There were no other significant interactions between the various dietary fiber and fat combinations. 

## 4. Discussion

The results of the present analysis in a large, prospective cohort of postmenopausal women do not support the results obtained in preclinical studies demonstrating that combinations of higher fiber and fat subtypes are associated with reduced risk of CRC. While there was a modest additional protective effect of either dietary component when intake of the other was low, this protective effect was attenuated with greater consumption in our study population. Intakes and ranges were very low such that only women in the highest quintile of fiber were consuming adequate intakes for this age group (21 g/day for women ages 51–70 years), with a very small percentage consuming high intakes [71]. Further, intakes of *n*-3 PUFA, and DHA and EPA, specifically, were substantially lower than the levels used in experimental diets. These factors may have prevented detection of an association.

It is generally thought that CRC risk could be reduced through dietary modification, including increased dietary fiber intake and reduced fat intake [3]. Dietary fiber mainly includes remnants of plant foods resistant to digestion by human enzymes and therefore arrives in the colon relatively intact, where it undergoes metabolism by gut microbiota. In the 2011 Colorectal Cancer Report, part of the Continuous Update Project, the World Cancer Research Fund/American Institute for Cancer Research Expert Panel classified evidence supporting consumption of fiber-containing foods and CRC protection as ‘convincing’ [72], noting that for every 10 g/day increase in fiber, there was a 10% decrease in CRC risk. Mechanisms hypothesized to explain how dietary fiber may reduce CRC include increased stool bulk and reduced intestinal transit time, resulting in reduced exposure to potential carcinogens; decreased secondary bile acids and subsequent generation of reactive oxygen species; and fiber fermentation by gut microbiota to short-chain fatty acids, particularly butyrate [73,74,75]. It is now well-documented that butyrate induces apoptosis in tumor cells through inhibition of HDAC and subsequent activation of the Fas receptor-mediated extrinsic death pathway [31,76,77,78,79]. Given the potential importance of fiber fermentation on CRC risk, consideration of fiber subtypes may be important; however, few studies have examined fiber subtype (i.e., soluble and insoluble, or more and less fermentable), and associations are similar, with both null [9,10] and inverse associations [80,81,82,83] reported. 

When evaluating total fiber and subtypes in the present study, CRC risk was reduced for all quintiles of increased consumption and a marginal linear trend was observed; however, most quintiles did not reach statistical significance. Inconsistencies across epidemiologic studies may be attributed in part to lower overall fiber intakes and narrow ranges of fiber intakes in Western populations which reduce statistical power [13]. Mean fiber intake in the U.S. is ~15 g/day [4]. Intakes at or above current recommended intakes (25 g/day for women and 38 g/day for men) [71] show robust protection against CRC with relative risks ranging from 0.72 to 0.90 [4,6,26]. Only 10% of our study population reported intakes of dietary fiber >25 g/day, thus our ability to detect a protective effect was compromised.

Overlaying the conflicting data observed with dietary fiber and CRC is a similar body of inconsistent literature on the association between subtypes of fat, particularly *n*-3 PUFA, and CRC risk. In addition to the anti-inflammatory effects promulgated by changes in the eicosanoid milieu, other putative actions of *n*-3 PUFA have been proposed. These include alterations in membrane fluidity and lipid raft composition, which may affect receptor signaling involved in proliferation and apoptosis [84], and modulation of oxidative stress [57,85]. As with dietary fiber, laboratory data consistently show reduced CRC risk with marine *n*-3 PUFA [58], while epidemiologic data are less convincing. Whereas two meta-analyses reported reduced CRC risk with *n*-3 PUFA from fish intake [86,87], two systematic reviews concluded that there is insufficient [15] or limited [7] evidence to suggest an association between long-chain *n*-3 PUFA intake and CRC risk. A more recent publication reported no overall association between *n*-3 PUFA and CRC risk among 123,529 individuals; however, among women in the same cohort, *n*-3 PUFA intake after CRC diagnosis had a protective effect on survival [88]. 

In the WHI population, we did not observe an association between any dietary fat subtypes and CRC risk. As with fiber, intakes of *n*-3 PUFA were very low; mean intakes were 1.4 g/day (range 0.1–10.4) for *n*-3, and 0.04 and 0.08 g/day (range 0–1.4 and 0–3.1, respectively) for EPA and DHA. While specific recommendations for *n*-3 PUFA intake have not been determined, the Food and Nutrition Board of the Institute of Medicine established adequate intake levels for alpha-linolenic acid (ALA), the precursor of long-chain *n*-3 PUFA, of 1.1 g/day for women over the age of 19 [71]. Only about half of our study population had intakes at that level. Furthermore, the Academy of Nutrition and Dietetics recommends a minimum intake of 0.5 g/day of combined EPA and DHA [89]. Less than 2% of the women in our study cohort reached an intake level above 0.5 g/day. 

Despite the inconsistent epidemiologic data for dietary fiber and fat, studies in preclinical animal models have shown that combinations of fiber and fat work synergistically to protect against colon cancer [58,59,60,61,62]. In a series of experiments, the effects of different types of fat (fish oil or corn oil; 15 g/100 g) and fiber (pectin or cellulose; 6 g/100 g) diets were tested on tumorigenesis and various other aspects of colonocyte physiology [58,90]. Both fish oil and pectin alone, relative to corn oil and cellulose feeding, resulted in a significantly lower proportion of animals with adenocarcinomas; however, the combination of fish oil and pectin led to an even greater reduction [58]. Additional work showed changes to the re-dox environment within rat colonocytes with a concomitant reduction in DNA damage [35,59,91]. Serving as the primary energy source for colonic epithelial cells, butyrate induces cellular reactive oxygen species (ROS) generation, creating a pro-oxidant environment [31]. At the same time, long chain *n*-3 PUFA from fish oil (e.g., DHA) incorporated into cell membranes, are susceptible to oxidation due to their high degree of unsaturation [33]. These physiological attributes of butyrate and DHA are relevant as lipid peroxidation can directly trigger the release of pro-apoptotic factors from mitochondria into the cytosol, through a p53-independent, calcium-mediated cell death pathway [33]. Given that butyrate independently induces apoptosis through an extrinsic, HDAC inhibition-mediated pathway, the combination of dietary constituents, and subsequent effects on intrinsic apoptotic pathways, would be expected to exponentially increase apoptosis. 

Dietary fiber and fatty acids, in combination, and risk of CRC in humans has only recently begun to be evaluated. In a large cohort study (*n* = 96,354) of Seventh Day Adventists, risk of CRC was reduced by 22% among all vegetarians combined compared to non-vegetarians, but protection was greatest among pescovegetarians who consume high amounts of both fiber and *n*-3 PUFA-containing fish (HR: 0.57; 95% CI: 0.40, 0.82) [92]. Furthermore, striking reciprocal changes in gut mucosal cancer risk biomarkers, the microbiome, and the metabolome were reported after African Americans were given a high-fiber (primarily in the form of resistant starch, including pectin), low-fat diet and rural Africans were given a high-fat, low-fiber Western-style diet [93]. Significantly increased butyrate production and suppressed secondary bile acid synthesis were also noted after the diet exchange to high-fiber, low-fat intake in African Americans [93]. 

An analysis in the Rotterdam prospective cohort (*n* = 4967) lends support to the hypothesis that higher intakes of fat and fiber are an important factor mediating the relationship between these dietary variables. An increased risk of CRC was observed with *n*-3 PUFA intake and dietary fiber intakes below the median, but not when fiber intakes were above [22]. It is noteworthy that the levels of fiber intakes were markedly lower (mean 26 g/day) relative to the intakes among pescovegetarians in the Seventh Day Adventist population (mean 40 g/day) and the African American intervention participants (mean 55 g/day), but higher than the intakes in our population (mean 16 g/day). Adding to the complexity of the relationship, when evaluated by food sources of *n*-3 PUFA in the Rotterdam study, increased CRC risk was restricted to intake from non-marine sources which contain ALA; there was no association when *n*-3 PUFA from marine-derived (e.g., EPA and DHA) sources were evaluated [22]. Contrary to these results, evaluation by *n*-3 PUFA source in a case-control study did not show any difference between non-marine and marine sources and interaction with dietary fiber. In this instance, greater protection of *n*-3 PUFA was found among individuals with lower dietary fiber intakes although overall intake was low (median 18.6 g/day) [94], consistent with our observation of the significant reduced risk of CRC among women with the lowest soluble fiber and highest DHA + EPA intakes. The authors speculated that high fiber intake may interfere with lipid absorption, offsetting potential beneficial effects of certain fatty acids [94]. 

While intakes in animal models do not directly translate to human intakes, the amount of fiber and fat used in preclinical experiments is comparable, as a proportion of intake, to what is currently recommended for humans. For example, the amount of fiber in the rat diets was 6% by weight, which is approximately equivalent to 30 g/day for humans [58]. This level of intake was only approached in the highest quintile of total fiber intake (>21.5 g/day). Similarly, all rat diets contained fat at 15% by weight and 30% of energy, with fish oil comprising approximately 1%–5% [58]. Women in WHI consumed 33% of their calories from fat on average, but less than 1% from *n*-3 PUFA and even less from combined DHA and EPA from fish oil (highest quintile >0.18 g/day). In the VITamins And Lifestyle (VITAL) cohort (*n* = 68,109), individuals using fish oil supplements on 4+ days/week for 3+ years experienced 49% lower CRC risk than non-users (HR = 0.51, 95% CI = 0.26–1.00; *p-trend* = 0.06) [17]. An average serving of oily fish (e.g., salmon or mackerel) provides 1.5–3 g of marine fish oil [95]. Thus, it may be difficult to achieve the levels of *n*-3 PUFA needed for a chemoprotective effect through diet alone. 

Strengths of this study include the large, well-characterized prospective cohort. As CRC was one of the primary outcomes of WHI, incident cases were expertly adjudicated and confirmed. Additionally, detailed information was collected on a wide range of exposures, including diet, with reliability subsequently assessed [65]. Finally, this is the largest prospective cohort to evaluate the interactions of subtypes of fiber and fat on CRC to date. 

As with all observational studies, there are limitations. First, we relied on self-report via FFQ to capture usual dietary intake, therefore we cannot rule out measurement error. FFQs are subject to lack of precision and inaccurate recall of dietary intake [96]. As dietary intakes were only assessed at baseline, it is possible that diets changed over time. However, we performed a sensitivity analysis using FFQ from women in the Dietary Modification comparison arm (the only group for which FFQ data from multiple time-points were collected) between years 0, 1, 3, 6, and 9, and found that correlations were 0.62 for fiber intake and 0.44 for total fat intake, suggesting that intake remained relatively stable during the follow-up. The WHI FFQ has been shown to yield nutrient estimates that are similar to those obtained from short-term dietary recall and recording methods, and test-retest reliability for most nutrients was high (e.g., FFQ estimates for total, saturated, and polyunsaturated fat variables were all within 10% of those from food records and 24-h recalls, although dietary fiber was slightly higher ~17%, and information on fat and fiber subtypes was not evaluated) [67]. Our fiber exposure also did not include the integration of dietary fiber supplement use, which is known to be higher with age and among women [97]. Lastly, measurement of the level of EPA/DHA incorporation in plasma or the tissue level was not conducted. It is widely known that the biological activities of these compounds depend on their bioavailability [98] and FFQ estimates only provide a surrogate for exposure.

Finally, while we adjusted for several potential confounders, we did not directly evaluate antioxidant content of the diet. Because the combination of butyrate and *n*-3 PUFA drive apoptosis via an oxidative-dependent mechanism, higher antioxidant intakes might antagonize this protective effect. In addition, there is some evidence that high antioxidants promote CRC in both preclinical [59,91] and epidemiologic [99,100,101] studies. Although mean intakes for several individual nutrients with antioxidant capacity (e.g., vitamins C, E, and beta-carotene) were very similar between cases and controls at baseline (<1% difference), total antioxidant capacity of dietary intakes was not considered. 

## 5. Conclusions

In summary, at the levels of dietary intake consumed by women in WHI, we did not find evidence of a protective association between increased subtypes of fiber and fat intakes in combination, and reduced risk of CRC. Our results for fat and fiber separately are in agreement with what has been reported previously, namely modest inverse associations with fiber, but no consistent trends across intakes. Given the biologic plausibility of a protective effect for fiber and *n*-3 PUFA, the robust results from preclinical models, and the mixed results in observational studies, controlled feeding trials with standardized intakes and consideration of the potential impact of the gut microbiome are urgently needed. 

## Figures and Tables

**Table 1:** Baseline characteristics, colorectal cancer risk factors, and dietary constituents for participants in the Women’s Health Initiative 1993–2010 ^1^.

Characteristic	Cases (*n* = 1952)	Non-Cases (*n* = 132,065)
Age, (years)	66 (6.9)	63 (7.3)
Height (cm)	162 (6.3)	162 (6.4)
Body mass index (kg/m^2^)	28 (5.7)	28 (5.5)
Race (%)		
White	85	84
Black	9	8
Other/Unknown ^2^	6	8
Education (% college graduate)	38	40
Screening colonoscopy (%)	49	51
Family history of CRC (%)	19	15
NSAID (% current use)	17	19
Alcohol use at baseline (g/day)	5 (11.3)	5 (10.8)
Never smokers (%)	49	50
Physical activity (MET-h/week) ^3^	12 (12.6)	13 (13.7)
Ever used post-menopausal Hormone therapy (%)	48	56
Dietary intake		
Total energy (kJ/day)	6766 (2671)	6787 (2642)
Total fiber (g/day)	16 (6.9)	16 (6.9)
Soluble fiber (g/day)	4.3 (1.8)	4.3 (1.8)
Pectins (g/day)	2.5 (1.2)	2.5 ( 1.2)
Insoluble fiber (g/day)	11.5 (5.1)	11.8 (5.1)
Total fat (g/day)	60 (32.7)	59 (32.0)
*n*-3 (g/day)	1.41 (0.8)	1.41 (0.8)
DHA + EPA (g/day)	0.12 (0.12)	0.13 (0.12)
*n*-6 (g/day)	10.8 (6.2)	10.7 (6.2)
Linoleic acid (g/day)	10.8 (6.2)	10.7 (6.1)
Linolenic acid (g/day)	1.3 (0.7)	1.3 (0.7)
Calcium (mg/day)	803 (439)	827 (453)
Folate (DFE, µg/day)	478 (206)	488 (207)
Red meat (g/day)	0.69 (0.57)	0.67 (0.55)

^1^ Means (Standard Deviation) unless otherwise specified; ^2^ Includes Hispanic, American Indian, Asian/Pacific Islander, Mixed races, and Refused; ^3^ Total metabolic equivalent-hours/week. CRC, colorectal cancer; DFE, dietary folate equivalents; DHA, docosahexaenoic acid; EPA, eicosapentaenoic acid; NSAIDs, non-steroidal anti-inflammatory drugs.

**Table 2:** Multivariate-adjusted hazard ratios and 95% CIs for the association of fiber with colorectal cancer in the Women’s Health Initiative (*n* = 134,017) 1993–2010.

Quintiles of Dietary Intake	No. Cases	Person-Years	Multivariate Adjusted HR (95% CI) ^1^
Total fiber (g/day)			
<10.3	420	302,828	1.00 (Reference)
10.3–13.6	426	312,546	1.00 (0.87, 1.15)
13.6–17.0	354	316,244	0.83 (0.71, 0.97)
17.0–21.5	372	318,893	0.87 (0.74, 1.03)
>21.5	380	318,844	0.90 (0.73, 1.10)
*p-trend*			0.09
Soluble fiber (g/day)			
<2.8	408	304,051	1.00 (Reference)
2.8–3.7	409	312,599	0.97 (0.84, 1.12)
3.7–4.6	361	316,928	0.85 (0.73, 1.00)
4.6–5.8	410	318,628	0.96 (0.81, 1.13)
>5.8	364	317,148	0.84 (0.69, 1.03)
*p-trend*			0.14
Insoluble fiber (g/day)			
<7.4	433	302,242	1.00 (Reference)
7.4–9.8	413	312,804	0.93 (0.81, 1.07)
9.8–12.3	357	316,721	0.81 (0.69, 0.94)
12.3–15.7	370	318,704	0.84 (0.72, 1.00)
>15.7	379	318,884	0.87 (0.72, 1.06)
*p-trend*			0.08
Pectin (g/day)			
<1.4	399	303,832	1.00 (Reference)
1.4–2.0	397	313,746	0.97 (0.84, 1.12)
2.0–2.6	379	316,302	0.92 (0.79, 1.06)
2.6–3.5	396	318,847	0.97 (0.83, 1.13)
>3.5	381	316,626	0.94 (0.80, 1.11)
*p-trend*			0.56

^1^ The following variables were included in both the multivariate and continuous models: total energy intake (continuous), age (continuous), body mass index (continuous), education (high school, technical school or some college, college graduate or post-graduate), family history of colorectal cancer (yes/no), history of colonoscopy (yes/no), current NSAID use (yes/no), alcohol intake (continuous), smoking history (never, former, current), physical activity (total metabolic equivalent-hours, continuous), ever use of hormone therapy (never, current/former), folate (DFE; µg/day, continuous), calcium (mg/day, continuous), and red meat intake (g/day, continuous), and study component (OS, CT) and CT randomization assignment and treatment arm. OS, observational study; CT, clinical trial; DFE, dietary folate equivalents; NSAIDs, non-steroidal anti-inflammatory drugs.

**Table 3:** Multivariate-adjusted hazard ratios and 95% CIs for the association of fat with colorectal cancer in the Women’s Health Initiative (*n* = 134,017) 1993–2010.

Quintiles of Dietary Intake	No. Cases	Person-Years	Multivariate Adjusted HR (95% CI) ^1^
Total fat (g/day)			
<33.1	345	309,005	1.00 (Reference)
33.1–45.6	432	318,884	1.20 (1.04, 1.39)
45.6–59.7	394	316,808	1.05 (0.90, 1.24)
59.7–80.6	381	316,629	0.98 (0.82, 1.18)
>80.6	400	313,067	0.98 (0.76, 1.27)
*p-trend*			0.44
*n*-6 PUFA (g/day)			
<5.9	375	309,046	1.00 (Reference)
5.9–8.1	405	314,292	1.02 (0.88, 1.18)
8.1–10.7	390	316,554	0.95 (0.81, 1.11)
10.7–14.6	394	317,021	0.92 (0.78, 1.09)
>14.6	388	312,441	0.84 (0.68, 1.05)
*p-trend*			0.10
*n*-3 PUFA (g/day)			
<0.80	401	308,532	1.00 (Reference)
0.80–1.09	389	314,521	0.94 (0.82, 1.08)
1.09–1.41	383	316,931	0.90 (0.78, 1.05)
1.41–1.90	377	316,759	0.87 (0.75, 1.03)
>1.90	402	312,611	0.90 (0.74, 1.09)
*p-trend*			0.16
DHA + EPA (g/day)			
<0.04	402	307,408	1.00 (Reference)
0.04–0.07	389	312,402	0.97 (0.84, 1.12)
0.07–0.11	393	315,998	0.98 (0.85, 1.13)
0.11–0.18	389	318,148	0.99 (0.85, 1.14)
>0.18	379	315,397	0.98 (0.84, 1.13)
*p-trend*			0.87

^1^ The following variables were included in both the multivariate and continuous models: total energy intake (continuous), age (continuous), body mass index (continuous), education (high school, technical school or some college, college graduate or post-graduate), family history of colorectal cancer (yes/no), history of colonoscopy (yes/no), current NSAID use (yes/no), alcohol intake (continuous), smoking history (never, former, current), physical activity (total metabolic equivalent-hours, continuous), ever use of hormone therapy (never, current/former), folate (DFE µg/day, continuous), calcium (mg/day, continuous), and red meat intake (g/day, continuous), and study component (OS, CT) and CT randomization assignment and treatment arm. OS, observational study; CT, clinical trial; DFE, dietary folate equivalents; DHA, docosahexaenoic acid; EPA, eicosapentaenoic acid; NSAIDs, non-steroidal anti-inflammatory drugs. PUFA, polyunsaturated fatty acids.

**Table 4:** Soluble fiber and DHA + EPA stratified by quintile of intake, and association with colorectal cancer in the Women’s Health Initiative (*n* = 134,017) 1993–2010.

		DHA + EPA (g/Day)					HR (95% CI) ^1^	*p*-Trend ^2^
Soluble fiber (g/day)		Q1	Q2	Q3	Q4	Q5		
	Q1	1.00	0.88	0.78	0.88	0.59	0.91	0.02
		(Ref) ^3^	(0.68, 1.13)	(0.59, 1.04)	(0.65, 1.20)	(0.40, 0.88)	(0.84, 0.98)	
	Q2	0.81	0.82	0.91	0.80	0.91	1.03	0.44
		(0.62, 1.06)	(0.63, 1.07)	(0.70, 1.18)	(0.60, 1.06)	(0.69, 1.23)	(0.96, 1.11)	
	Q3	0.71	0.84	0.73	0.65	0.79	0.99	0.70
		(0.53, 0.96)	(0.63, 1.11)	(0.55, 0.97)	(0.48, 0.87)	(0.59, 1.06)	(0.91, 1.06)	
	Q4	0.76	0.86	0.87	0.90	0.77	1.00	0.96
		(0.56, 1.05)	(0.65, 1.15)	(0.66, 1.15)	(0.68, 1.18)	(0.57, 1.02)	(0.93, 1.07)	
	Q5	0.70	0.53	0.73	0.81	0.80	1.08	0.07
		(0.49, 1.01)	(0.37, 0.78)	(0.53, 1.00)	(0.60, 1.08)	(0.60, 1.08)	(1.00, 1.17)	
HR (95% CI) ^1^		0.97	0.90	1.02	0.99	1.00		
		(0.89, 1.08)	(0.81, 1.00)	(0.92, 1.13)	(0.89, 1.09)	(0.89, 1.11)		
*p-trend* ^2^		0.62	0.05	0.72	0.80	0.95		

^1^ HR and 95% CI across quintiles of soluble fiber and DHA + EPA; ^2^ *p*-trend values from linear interactions across each row and column. The following variables were included in the model: total energy intake (continuous), age (continuous), body mass index (continuous), education (high school, technical school or some college, college graduate or post-graduate), family history of colorectal cancer (yes/no), history of colonoscopy (yes/no), current NSAID use (yes/no), alcohol intake (continuous), smoking history (never, former, current), physical activity (total metabolic equivalent-hours, continuous), ever use of hormone therapy (never, current/former), folate (µg/day, continuous), calcium (mg/day, continuous), and red meat intake (g/day, continuous), and study component (OS, CT) and CT randomization assignment and treatment arm; ^3^ the combination of the first quintiles for soluble fiber and DHA + EPA is the reference group for all comparisons within quintiles. OS, observational study; CT, clinical trial; DFE, dietary folate equivalents; DHA, docosahexaenoic acid; EPA, eicosapentaenoic acid; NSAIDs, non-steroidal anti-inflammatory drugs. PUFA, polyunsaturated fatty acids.
